# Agreement of BNP and NT-proBNP and the influence of clinical and laboratory variables

**DOI:** 10.1590/S1679-45082013000300003

**Published:** 2013

**Authors:** Milena Novaes Cardoso Curiati, Odilson Marcos Silvestre, Lucas José Tachotti Pires, Sandrigo Mangini, Philippe Vieira Pires, Fabio Antonio Gaiotto, André Micheletto Laurino, Paulo Manuel Pêgo-Fernandes, Carlos Eduardo dos Santos Ferreira, Fernando Bacal

**Affiliations:** 1 Hospital Israelita Albert Einstein, São Paulo, SP, Brazil; 2 Instituto do Coração, Hospital das Clínicas, Faculdade de Medicina, Universidade de São Paulo, São Paulo, SP, Brazil

**Keywords:** Natriuretic peptide, Heart failure

## Abstract

**Objective:**

To correlate the serum levels of B type natriuretic peptide and the N-terminal fraction of the pro-B type natriuretic peptide, as well as to analyze the influence of age, obesity, renal failure, left ventricle ejection fraction, diastolic dysfunction, and anemia on serum levels of both markers.

**Methods:**

An observational study in which the agreement was compared between these markers in consecutive samples of 138 patients. For the correlation, Pearson's test was used, and p<0.05 was considered statistically significant.

**Results:**

A linear association was observed between the B type natriuretic peptide and N-terminal fraction of the pro-B type natriuretic peptide (r = 0.907; p<0.001). When evaluating the categorized measurements as normal and altered, there was good agreement, with 90.6% of agreement classifications (p<0.001) in which altered values of the N-terminal fraction of the pro-B type natriuretic peptide and normal values of the B type natriuretic peptide represented 8.7% of the total; the opposite situation represented 1% of the total. Assessment of the influence of the clinical and laboratorial factors on the levels of natriuretic peptides showed that they rise according to age, but that they fall as the ejection fraction increases. Patients with anemia (p<0.001) or with renal failure (p=0.007) had higher values of both markers. There was no association between obesity and the B type natriuretic peptide.

**Conclusion:**

There was satisfactory agreement between the B type natriuretic peptide and the N-terminal fraction of the pro-B type natriuretic peptide. Age, creatinine levels, and hemoglobin, as well as ventricular function, influence the serum levels of both natriuretic peptides.

## RESUMO

**Objetivo:**

Correlacionar os níveis séricos do peptídeo natriurético tipo B e da fração N-terminal do pró-peptídeo natriurético tipo B, além de analisar a influência de idade, obesidade, insuficiência renal, fração da ejeção do ventrículo esquerdo, disfunção diastólica e anemia nos níveis séricos de ambos os marcadores.

**Métodos:**

Estudo observacional, no qual se comparou a concordância entre esses marcadores em amostras consecutivas de 138 pacientes. Para a correlação, utilizou-se o teste de Pearson e foi considerado estatisticamente significante p<0,05.

**Resultados:**

Observou-se associação linear entre peptídeo natriurético tipo B e fração N-terminal do pró-peptídeo natriurético tipo B (r=0,907; p<0,001). Ao se avaliarem as medidas categorizadas como normais e alteradas, encontrou-se boa concordância, com 90,6% de classificações concordantes (p<0,001), sendo que valores alterados da fração N-terminal do pró-peptídeo natriurético tipo B e normais de peptídeo natriurético tipo B representaram 8,7% do total, e o contrário representou 1% do total. A avaliação da influência dos fatores clínicos e laboratoriais nos níveis dos peptídeos natriuréticos mostrou que eles se elevam de acordo com a idade, mas que diminuem conforme aumenta a fração de ejeção. Pacientes com anemia (p<0,001) ou com insuficiência renal (p=0,007) apresentaram valores maiores de ambos os marcadores. Não houve associação entre obesidade e peptídeo natriurético tipo B.

**Conclusão:**

Houve concordância satisfatória entre peptídeo natriurético tipo B e a fração N-terminal do pró-peptídeo natriurético tipo B. Idade, níveis de creatinina e hemoglobina, assim como função ventricular, influenciam os níveis séricos de ambos os peptídeos natriuréticos.

## INTRODUCTION

Natriuretic peptides are biomarkers that currently have ample use in difference scenarios of medical practice. Both B type natriuretic peptide (BNP) and the inactive N-terminal chain of the pro-BNP (NT-proBNP) have good accuracy in the differential diagnosis of dyspnea in emergency rooms^(1,2)^, both in a suspected diagnosis of heart failure (HF) in primary care^(3)^, and in a prognostic evaluation of patients with acute HF^(4)^. In addition to increasing diagnostic accuracy in the clinical examination and chest X-ray, cost analyses show that both natriuretic peptides are cost-effective in the investigation of HF^(5,6)^.

BNP and NT-proBNP result from cleavage of pro-BNP and are secreted by ventricular myocytes in response to volume or pressure overloads, in which BNP is the biologically active peptide with a vasodilating and natriuretic effect, antagonizing the vasoconstriction effect of the renin-angiotensin-aldosterone system^(7)^.

The influence of clinical and laboratorial factors on the serum level of natriuretic peptides has been discussed in literature. Thus, both BNP and NT-proBNP have high serum levels in the presence of advanced age, female gender, anemia, and renal dysfunction^(8,9)^. Inversely, obesity determined lower levels of these markers^(10)^. Prior studies showed different impacts of these factors in dosing one or the other natriuretic peptide. However, in most of these studies, there was no simultaneous evaluation of both markers, precluding the assessment, based on the results, of a similar impact of these clinical variables on both the levels of BNP and NT-proBNP in the same population. As an example, some authors suggested that NT-proBNP would suffer a greater influence of the altered renal function than would BNP^(11)^. Additionally, since dosing of both biomarkers is commercially available, the issue as to equivalence and the comparison between the two is clinically relevant. In the Brazilian population, there are no current studies directly comparing the performance of the more recent diagnostic kits of both the natriuretic peptides or on the interference of clinical factors in this dosing.

## OBJECTIVE

The present study had the objective of correlating the serum levels of B type natriuretic peptideand the N-terminal fraction of the pro-B type natriuretic peptide, besides analyzing the impact of the variables age, obesity, renal failure, left ventricle ejection fraction, and anemia in the serum levels of both markers.

## METHODS

An observational retrospective study in which the levels of BNP and NT-proBNP in the consecutive samples of 138 patients of the *Hospital Israelita Albert Einstein,* in Sao Paulo, were collected during the period from January 20^th^ to 31^st^, 2012, and analyzed in the laboratory of the same hospital. Electronic medical charts were analyzed to collect the following data: age, gender, weight, height, left ventricle ejection fraction determined by echocardiography, dosing of hemoglobin, creatinine, and urea. The study was approved by the local Ethics Committee, under number 353,939.

BNP dosing was done using the Alere Triage™ BNP (Alere, San Diego, California, USA) kit, a fluorescence immunoassay device. In the analysis, the concentrations of BNP are directly proportional to the fluorescence detected by measurements made with the Alere Triage™ Meter. The samples were collected in EDTA-anticoagulated test tubes and the analyses were performed within 7 hours after the collection, as per instructions of the test's manufacturer. Values were given in picograms per milliliter (pg/mL).

Dosing of NT-proBNP was done with the VITROS™ NT-proBNP (Ortho-Clinical Diagnostics, UK) kit. The plasma samples were EDTA-anticoagulated, as per the manufacturer's recommendation, and analyzed by immunometric immunoassay technique, which involves the simultaneous reaction of NT-proBNP present in the sample with a biotinylated antibody (ewe anti-BNP/proBNP) and an antibody conjugate marked with horseradish peroxidase. Values were given in picograms per milliliter (pg/mL).

For definition of diseases, anemia was considered in the presence of hemoglobin<12.0g/dL; renal failure was diagnosed based on the calculation of a glomerular filtration rate<60 mL/min and obesity in the presence of BMI≥30kg/m^2^. The levels of BNP were considered increased when≥100pg/dL, whereas NT-proBNP was defined as abnormal when≥300pg/dL.

In the statistical analysis, qualitative data were described by absolute frequency and percentage, and quantitative data by median, 1^st^ and 3^rd^ quartiles, minimum and maximum, due to asymmetry. The values of BNP and NT-proBNP were transformed logarithmically for analysis because of the asymmetry of the values. The association between BNP and NT-proBNP was evaluated by dispersion graph and Pearson's coefficient of correlation. For categorized measurements, agreement was assessed by double entry tables and Cohen's kappa coefficient of agreement. To evaluate the relationship among variables seen with BNP and NT-pro-BNP normal multiple linear regression was used. Analyses were performed with the aid of the Statistical Package for the Social Sciences (SPSS) program, version 17.0 (Chicago, USA). Values of p<0.05 were considered statistically significant.

## RESULTS

**Figure 1:**
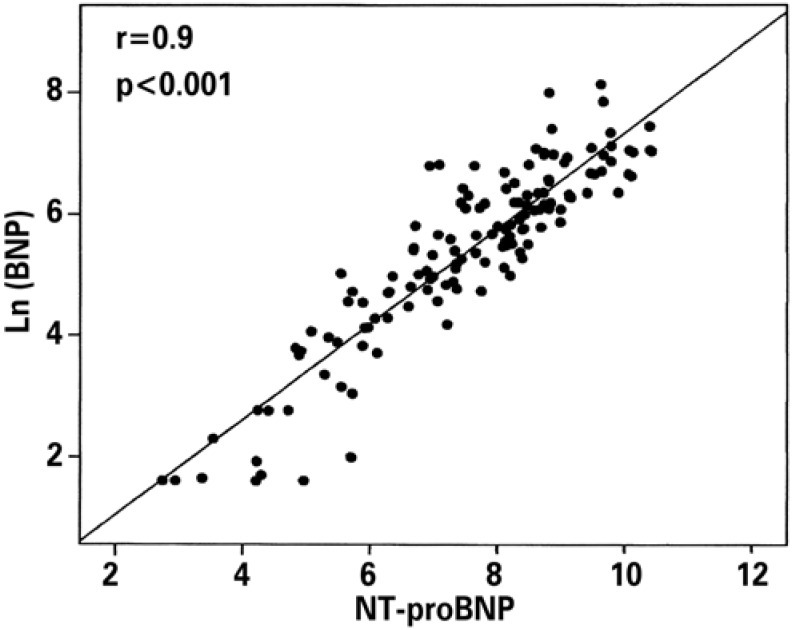
Correlation between B type natriuretic peptide and the N-terminal pro-BNP fraction

Of the 138 patients, 88 (63.8%) were male, with a median age of 81 years. Levels of BNP and NT-proBNP had a median of 287pg/mL (5.0-3,410) and 2,600 (15.5-33,700), respectively (Table 1). A linear association was observed between BNP and NT-proBNP (r=0.907; p<0.001) (Figure 1). In evaluating the categorized measurements as normal and altered, 90.6% of the classifications were in agreement (Cohen's kappa coefficient 0.699; p<0.001), and the altered values of NT-proBNP and normal values of BNP represented 8.7% of the total; inversely, (altered values of BNP and normal values of NT-proBNP) represented 1% of the total (Table 2).

**Table 1:** Value of the B type natriuretic peptide, the inactive N-terminal pro-BNP chain, and clinical and laboratorial parameters

	Median	Minimum	1st quartile	3rd quartile	Maximum
NT-proBNP (pg/mL)	2,600.0	15.5	738.0	6,230.0	33,700.0
BNP (pg/mL)	287.0	5.0	113.0	612.0	3,410.0
Age (years)	81	4	69	86	98
Creatinine (mg/dL)	1.14	0.39	0.82	1.60	6.60
Urea (mg/dL)	63	12	45	90	253
Weight (Kg)	74.5	16.0	63.0	87.0	144.0
Height (meters)	1.67	1.42	1.62	1.80	1.90
BMI (kg/m2)	26.6	13.7	23.0	30.1	41.3
LVEF (%)	60	18	43	65	79
Hemoglobin (g/dL)	11.6	7.6	9.6	12.9	17.5

NT-proBNP: N-terminal fraction of pro-BNP; BMI: body mass index; LVEF: left ventricle ejection fraction.

**Table 2:** Agreement between the B type natriuretic peptide and the inactive N-terminal pro-BNP chain. Comparison between normal and altered values

			BNP		Total
			Normal	Altered	
NT-proBNP	Normal	n	20	1	21
		%	14.5	0.7	15.2
	Altered	n	12	105	117
		%	8.7	76.1	84.8
Total		n	32	106	138
		%	23.2	76.8	100.0

NT-proBNP: N-terminal fraction of pro-BNP; BNP: B type natriuretic peptide.

Assessment of the influence of the clinical and laboratorial variables on the concentration of BNP and NT-proBNP showed that, the more advanced the age, the greater the value of BNP or NT-proBNP (p<0.05) and that the greater the ejection fraction, the lower the value of BNP or NT-proBNP (p<0.05). Patients with anemia or renal failure showed higher values of BNP, as can be seen on table 3. There was no association between natriuretic peptides and obesity.

**Table 3:** Correlation between the B type natriuretic and the inactive N-terminal pro-BNP chain with the clinical and laboratorial variables

	BNP (95% CI)	p value	NT-proBNP (95% CI)	p value
Age	0.176 (0.003;0.027)	0.018	0.228 (0.009;0.035)	0.002
LVEF	-0.224 (-0.039;-0.008)	0.003	-0.264 (-0.049;-0.015)	<0.001
Obesity (BMI>30)	-0.104 (-0.833;0.131)	0.152	-0.074 (-0.82;0.245)	0.287
Anemia (yes)	0.436 (0.851;1.715)	<0.001	0.451 (1.056;2.01)	<0.001
Renal failure (yes)	0.206 (0.174;1.058)	0.007	0.214 (0.250;1.225)	0.003

95% CI: 95% confidence interval; NT-proBNP: N-terminal pro-BNP fraction; LVEF: left ventricle ejection fraction; BMI: body mass index.

LN(BNP): neperian logarithm of BNP; LN(NT-proBNP): neperian logarithm of NT-proBNP.

## DISCUSSION

The present study evaluated the agreement between the natriuretic peptides most often applied in medical practice for the diagnosis of HF, besides investigating the interference of clinical and laboratorial factors in the serum levels of these biomarkers. The findings of the study show that there is a good level of agreement between them and that, additionally, they are equivalent as to the influence of age, systolic dysfunction, anemia, and renal failure.

As is true in this study, other studies that analyze the agreement between BNP and NT-proBNP showed a strong correlation between them. When there was disagreement, it was evident especially in patients with decreased renal function (creatinine clearance<60mL/ min), in which NT-proBNP suffered greater interference, showing altered results that disagreed with the results within normal range of BNP^(12)^. Similar to preceding authors, this study showed that when there are differing results in the comparison between biomarkers, most often there is an elevation of NT-proBNP with normal values of BNP. In any case, the high correlation between the levels of these markers makes both of them suitable for the diagnostic evaluation of patients with dyspnea and for the prognostic evaluation of situations that evolve with ventricular overload, as in patients with HF and pulmonary thrombosis, as long as the differences in their levels of normality and range of elevation are observed for the same clinical condition, a situation caused by different metabolism times that occur with each one of these peptides.

The influence of clinical and laboratorial factors on the levels of natriuretic peptides has been previously studied. Some authors showed that there is an increase both of BNP and of NT-proBNP with the increase in age, with worsening of renal function and a drop in hemoglobin^(8,9)^. On the other hand, obese patients would have the lowest levels of both the natriuretic peptides^(10)^, as well as expected elevated levels of these markers in the presence of depressed ventricular function^(1)^. The reasons that lead to the relation between age and the increased levels of BNP are not yet clear. Hypotheses that suggest alterations of diastolic function or other cardiac structural alterations related to aging have not yet been confirmed in prior analyses^(9)^. It is possible that this increase is the result of microstructural alterations in the atrial and ventricular myocardial that cannot be verified by means of the diagnostic methods studied until now within this context. Similarly, the studies that showed an inverse relation between the body mass index (BMI) and the levels of BNP suggested a possible increase in the degradation of the adipose tissue peptide as an explanation for this relation^(10)^. In this set of cases, however, there was no correlation between weight or BMI and the values of the biomarkers analyzed, possibly due to the limited size of the sample or because of an effect of lesser magnitude of this factor (systematic error type β).

Anemia, on the other hand, despite being common in patients with HF and its association with severity of the disease, in prior studies presented with BNP and NT-proBNP in a manner independent from the severity of patients' condition^(8)^. One hypothesis for this relation would involve a possible increase in plasma volume present in patients with anemia, leading to greater stimulation for the release of natriuretic peptides by the myocardium^(8)^. The relation between the increased levels of these peptides and renal failure, on the other hand, would be related to the decrease in clearance of these biomarkers, which in part would occur through the kidneys.

Therefore, we demonstrated herein that both BNP and NT-proBNP are influenced in an equivalent manner by these factors, highlighting the idea that an individual interpretation is necessary of the levels of natriuretic peptides, always considering the other clinical aspects associated with the numeric value of the test in order to increase diagnostic accuracy of these peptides.

## CONCLUSION

There was a satisfactory agreement between the serum levels of B type natriuretic peptide and the N-terminal fraction of the pro-B type natriuretic peptide. Both the natriuretic peptides suffered interference of age, ventricular systolic function, and levels of hemoglobin and creatinine. These findings do not limit the use of these markers, but bring to light the need for interpretation of their serum levels in a manner integrated with clinical parameters, with the goal of optimizing the success of the treatments implemented.
